# An efficient smart phone application for wheat crop diseases detection using advanced machine learning

**DOI:** 10.1371/journal.pone.0312768

**Published:** 2025-01-08

**Authors:** Awais Amir Niaz, Rehan Ashraf, Toqeer Mahmood, C. M. Nadeem Faisal, Muhammad Mobeen Abid

**Affiliations:** Department of Computer Science, National Textile University, Faisalabad, Pakistan; Indian Agricultural Research Institute, INDIA

## Abstract

Globally, agriculture holds significant importance for human food, economic activities, and employment opportunities. Wheat stands out as the most cultivated crop in the farming sector; however, its annual production faces considerable challenges from various diseases. Timely and accurate identification of these wheat plant diseases is crucial to mitigate damage and enhance overall yield. Pakistan stands among the leading crop producers due to favorable weather and rich soil for production. However, traditional agricultural practices persist, and there is insufficient emphasis on leveraging technology. A significant challenge faced by the agriculture sector, particularly in countries like Pakistan, is the untimely and inefficient diagnosis of crop diseases. Existing methods for disease identification often result in inaccuracies and inefficiencies, leading to reduced productivity. This study proposes an efficient application for wheat crop disease diagnosis, adaptable for both mobile devices and computer systems as the primary decision-making engine. The application utilizes sophisticated machine learning techniques, including Decision Tree (DT), Random Forest (RF), Support Vector Machine (SVM), and AdaBoost, combined with feature extraction methods such as Count Vectorization (CV) and Term Frequency-Inverse Document Frequency (TF-IDF). These advanced methods collectively achieve up to 99% accuracy in diagnosing 14 key wheat diseases, representing a significant improvement over traditional approaches. The application provides a practical decision-making tool for farmers and agricultural experts in Pakistan, offering precise disease diagnostics and management recommendations. By integrating these cutting-edge techniques, the system advances agricultural technology, enhancing disease detection and supporting increased wheat production, thus contributing valuable innovations to both the field of machine learning and agricultural practices.

## 1. Introduction

The agriculture sector in Pakistan is the backbone of economic growth and it was the 7th largest wheat-producing country in 2013 because it has a large geographical area for agriculture [1]. It has the oldest and largest irrigation system in the entire South Asian region, according to the FAO (Food and Agriculture Organization of the United Nations) [2, 3]. In terms of nominal gross domestic product (GDP), the country’s economy ranked 23rd in the world (purchasing power parity) [4].

Pakistan now stands as a top 10 producer of cotton, wheat, sugarcane, dates, mango, and kinnow (oranges), as well as a top 10 producer of rice. Major crops (wheat, rice, cotton, and sugar cane) contribute approximately 4.9% of overall GDP, while other crops contribute 2.1% [4], with the decline in wheat production from number 7^th^ to 10^th^, the agriculture sector is facing many problems and difficulties in wheat crop production [1, 3]. This decline is partly attributed to limitations in current disease detection methods, which are often inaccurate and inefficient, contributing to substantial losses in yield and economic value.

The low levels of wheat production can be exacerbated by several factors, including inadequate early disease detection, outdated cultivation methods, and limited knowledge about modern agricultural practices. Disease detection is particularly critical, as it can reduce annual global wheat production by 15%–20% [5]. Agriculture extension services, though prominent in Punjab, are hindered by the lack of trained agriculturists and modern machinery and equipment [3]. Efficient early disease detection could significantly enhance crop management and protection methods, but the lack of trained agriculturists and state-of-the-art machinery and equipment. To overcome the problems, early disease detection in various crops as well as in wheat crops can benefit farmers for batter management and apply different methods to protect the crop. [3]. Furthermore, disease detection in rural areas is often ineffective and inefficient due to the shortage of agriculturists, affecting approximately 70% of the country’s rural population [1]. Wheat plant diseases are challenging for farmers to identify, requiring them to manually inspect large fields or rely on agricultural experts, which is time-consuming and labor-intensive [6].

Recent advancements in computer technology, particularly in human-computer interaction and artificial intelligence (AI), have opened new avenues for improving disease detection. Intelligent systems utilizing AI-based and Computer Vision (CV) methods can assist farmers in identifying wheat diseases more efficiently. Despite these advancements, many existing systems face challenges due to the limited scope of disease datasets, which restrict their ability to detect a wide range of wheat diseases accurately. To address these challenges, there is a growing need for comprehensive datasets and robust machine learning (ML) systems that can operate effectively across diverse field conditions [7–11].

In this study, a state-of-the-art (SOTA) and new dataset is introduced that has symptom-based text data for wheat diseases. The information in the database comes from a wide variety of wheat farms, internet, literature, and the University of Agriculture Faisalabad in the province of Punjab, Pakistan. This research focused on fourteen types of diseases black stem rust, leaf rust, stripe rust, loose smut, flag smut, complete bunt, partial bunt, ear cockle, tundo, black point complex, common bunt, sooty head molds, stagonospora nodorum blotch, and barley yellow dwarf. In text-based datasets, cases of these problems include, have been narrowly studied, which offers a general method for diagnosing diseases affecting wheat crops. The proposed system efficiently identifies wheat diseases based on text data using the ML model. Wheat disease detection is reviewed by field experts. This helped in two ways: (1) The accuracy and precision of the ML models have been improved, and (2) it generated useful agricultural information in the form of rules and disease categories that can be incorporated into this study. Finally, a performance-based comparison of the proposed system to the current state-of-the-art approaches in the literature. In this study, an intelligent system for diagnosing wheat crop diseases has been proposed.

This study presents a system for wheat disease detection based on ML techniques. Through a comprehensive investigation of different feature extraction and classification algorithms, to achieve high accuracy with 14 different diseases of wheat crops. The primary contributions of this study are outlined below:

1. This study concentrates on a key crop in Pakistan, namely wheat. An upgraded text-based dataset for wheat diseases is generated, compiled from diverse sources in Pakistan, and incorporating state-of-the-art information from [1]. This study emphasizes 14 specific wheat diseases.
2. The system provides management for identifying fourteen prevalent wheat diseases through an application serving as the decision-making engine in the backend. Additionally, an application has been developed to function as the user interface in the front end.
3. The validation of the diagnostic of various wheat diseases is additionally confirmed by domain experts, contributing to the enhanced accuracy of the system.
4. The effectiveness of two distinct vectorization methods, namely count vectorization and TF-IDF is assessed in terms of performance that is accuracy, precision, recall, and f1 score.
5. Fine-tuned the machine learning model through hyperparameter tuning, adjusting three specific hyperparameters (Maximum Depth, Minimum Samples Split, and Minimum Samples Leaf) when employing CART DT as well as in Random Forest. This adjustment aims to select the optimal model, preventing overfitting, a resource-intensive process.
6. A comparative assessment was carried out to evaluate ML techniques for recognizing wheat diseases. The proposed system demonstrated an accuracy of 99% in classifying wheat diseases. Owing to its robust generalization and notable recognition rate, the proposed system is suitable for deployment in diverse real-time industrial applications.

The subsequent sections of this paper are organized as follows: **Section 2** Literature review pertinent literature on wheat disease classification using machine learning. **Section 3** details the study’s proposed research methodology, encompassing data collection, data preparation, feature extraction, and machine learning model selection and evaluation. **Section 4** presents the study’s results and discussion, including the performance of four ML algorithms and the influence of hyperparameter tuning. **Section 5** concludes the paper by summarizing the key findings and suggesting directions for future research in wheat disease classification using machine learning.

## 2. Literature review

Artificial intelligence (AI) has revolutionized numerous fields by embedding human-like abilities such as learning, reasoning, and perception into software. This advancement allows computers to perform tasks that were once exclusively handled by humans. With the increase in computing power, access to large datasets, and the development of advanced AI algorithms, AI is now utilized in a variety of domains. These applications range from finger vein recognition [12] and diabetic retinopathy detection [13–17] to RNA engineering [18, 19], cancer detection [20–22], biomathematical problems [13, 23, 24], and smart agriculture [25].

The agriculture industry is severely impacted whenever significant crops are attacked by pests. Wheat, maize, rice, and other food crops are especially vulnerable to disease and need to be protected and managed effectively. The agricultural sector has suffered economically over the last couple of decades as a result of a global reduction in crop yield caused by various diseases. Wheat is susceptible to many different diseases, but some of the worst are loose smut, yellow/stripe rust, flag smut, and black stem rust. Various researchers have made contributions to distinct facets of precision agriculture [26]. Precise and early detection of plant diseases is vital for enhancing agricultural productivity sustainably. Historically, human specialists have been essential for identifying plant anomalies resulting from diseases, pests, nutrient deficiencies, or adverse weather conditions [27]. Fast and precise recognition of wheat leaf diseases and their severity is advantageous for the exact prevention and management of such diseases [28].

The main issue in most countries, including Pakistan, is low crop production as a result of late and faulty disease detection [29]. The main cause of low crop yield in developing countries, including Pakistan, is incorrect and late disease diagnosis [29, 30]. In Pakistan, Some of the prominent diseases are as follows: loose smut, yellow or stripe rust, flag smut, and black stem rust [31]. Research in this area has recently demonstrated sizable economic advantages [32, 33]. It demonstrates that agricultural productivity is increasing. Many successful expert systems in a variety of fields, such as economics, healthcare, education, weather forecasting, market trends, and different kinds of planning activities, have been created as a result of technology adaptation [34–37]. Statistics regarding Pakistani smartphone usage patterns have been revealed by the Pakistan Advertiser Society [38]. In Pakistan, 72% of people use smartphones. 68% of those who use smartphones are Android users. 60% of users have multiple cell phones. The country’s 3G and 4G networks have proliferated widely, fueling the rapid expansion of the smartphone market. So, various strategies and applications specifically disease diagnosis systems have been put forth in literature to help the agriculture sector. In [39], a data acquisition framework based on the traditional waterfall model proposed to collect crop disease data.

In [40], the authors discussed various approaches and methodologies used in the CLAES (Central Laboratory for Agricultural Expert Systems) Egypt to create expert systems. CLAES has also researched Egyptian rice disease classification rules. To derive classification rules and assess them in comparison with NN (Neural Networks) outcomes, the C4.5DT (Decision Tree) algorithm utilized by the authors. The performance of the CITEX (Citrus Expert System) and the CUPTEX (Cucumber Expert System) has been defined as an agricultural training tool by [41]. CUPTEX and CITEX are divided into four sub-systems with the names irrigation, fertilization, treatment, and verification. Performance improvements are seen after various experiments. The early diagnosis of fruit disease has been achieved using a variety of data mining techniques [42]. Spore traps and automatic meteorological stations are used, respectively, to gather weather and spore data. The percentage of instances that are successfully classified, the SD (Standard Deviation), the MAE (Mean Absolute Error), and the RAE (Relative Absolute Error) have all been calculated using various data mining approaches, such as J48, SMO (Sequential Minimal Optimization), and Zero-R. The fruit infection prediction model trained using the J48 algorithm, which successfully identified 90% of the total occurrences [42].

Back-propagation neural networks have been used in China to create an “intelligent web based system for diagnosis” for the management of cotton diseases [43]. Eight distinct types of cotton diseases have been used to evaluate this technology. For the identification of diseases in the oilseed crops soybean, peanuts, and rapeseed mustard, fuzzy logic has been employed in India [44]. For the wheat crop, [45] suggests an expert system that makes inferences based on rules. This system facilitating two-way cloud-based communication, disease recognition algorithms, and automated services for crops developed by [46]. A variety of parameter settings have been used to assess performance. To aid farmers in identifying plant illnesses, classification algorithms are introduced in [47]. It can also provide advice on how to treat various diseases. An image processing-based method for plant disease diagnosis is proposed in [48]. Clustering techniques are applied to the leaf image to segment out the damaged area. The disease is estimated by a fuzzy logic-based classifier based on attributes of the affected part. To control agricultural diseases, [49] give a review of existing expert systems.

In this study, there are few viable theoretical frameworks, and substantial work remains on the practical side of disease detection. For instance, diagnosing fruit diseases using a rule-based expert system has been proposed in [50]. In [51], a graphical and descriptive approach for locating plant illnesses is suggested and prototyped. Additionally, [52] propose a knowledge management and acquisition system for storing and managing crop disease data, which may serve as a basis for future diagnoses. A web-based application utilizing a rule promotion technique for diagnosing illnesses in oilseed crops is presented in [53]. Meanwhile, [43] offer a neural network-based solution for disease diagnosis in Chinese cotton using a backpropagation algorithm. [54] introduces new features in a mobile app for Windows Phones that employs image processing methods for early plant disease detection. In rural Africa, [55] present a smartphone-based app with a focus on diagnosing banana and tuber infections. Despite advancements in machine learning (ML) for disease detection, existing methods exhibit notable shortcomings, such as limited dataset diversity, inadequate handling of noisy or incomplete data, and reduced robustness across varying field conditions. For instance, while the mobile application Plantix provides disease diagnosis through leaf images and offers advice for extension professionals and farmers [56], its primary focus on visual symptom analysis does not address dataset limitations or adaptability to diverse conditions. Similarly, deep neural networks discussed in [57] face challenges with data variability and generalization. Image processing methods for early disease detection, as outlined in [58], may struggle with accuracy and applicability across a broad range of diseases. A literature review in [59] highlights smartphone sensors in agriculture but notes practical deployment issues. In response to these gaps, this study introduces a system that leverages a curated text-based dataset specifically compiled from agricultural research publications, extension manuals, and expert annotations [1, 3]. While the dataset size is relatively small, it has been meticulously curated to include the most prevalent and impactful wheat diseases. The focus on quality over quantity ensures that the dataset captures the essential characteristics necessary for accurate disease detection. Additionally, advanced machine learning techniques—such as Decision Trees (DT), Random Forest (RF), Support Vector Machines (SVM), and AdaBoost—are employed. These are combined with feature extraction techniques like Count Vectorization (CV) and Term Frequency-Inverse Document Frequency (TF-IDF). These advanced methods enable the model to achieve up to 99% accuracy in diagnosing fourteen prevalent wheat diseases. Although the dataset size might not be extensive, the robust preprocessing techniques and careful selection of features ensure model stability and reliability, making a significant contribution to wheat disease detection.

## 3. Proposed research methodology

**Fig 1:**
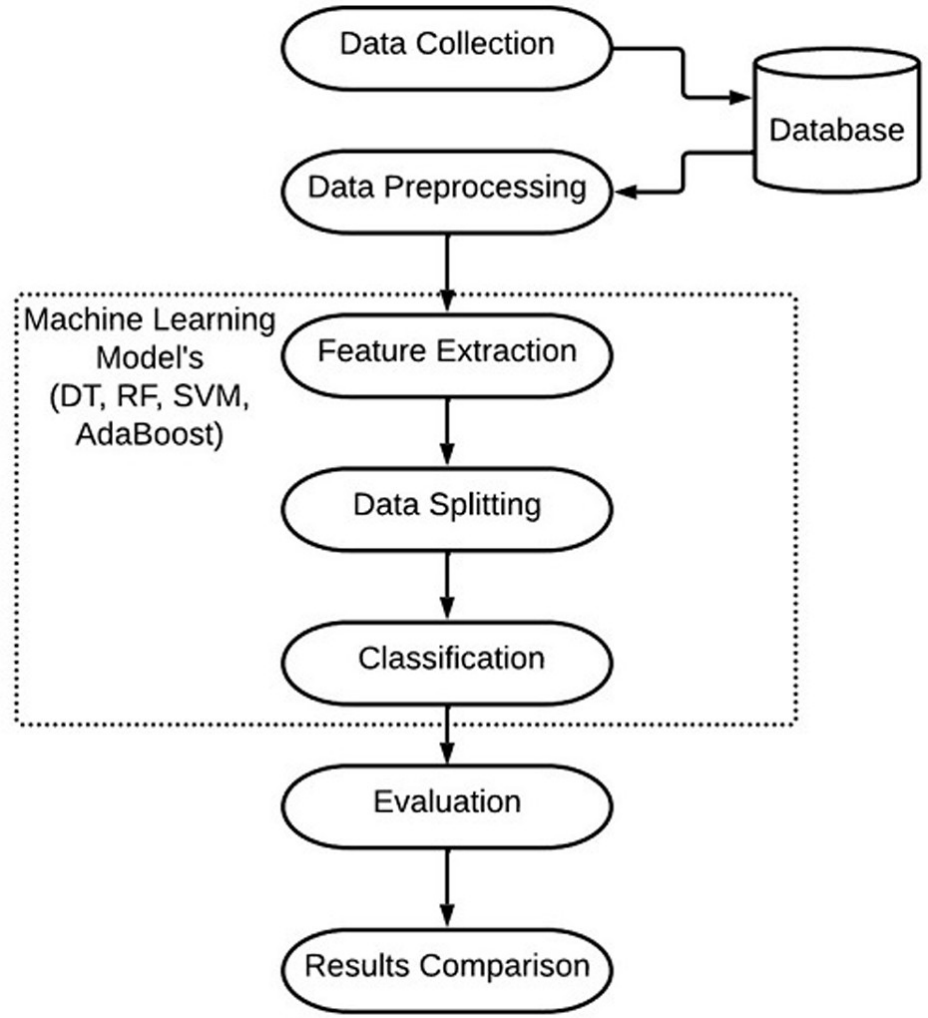
Proposed methodology for wheat crop disease recognition.

In this research, the wheat crop has been selected for implementation of the proposed method. Using the verification of domain experts, the implementation of this methodology has enabled the detection and classification of wheat diseases. The process flow of the suggested method is illustrated in Fig 1. Textual information has been provided by the domain expert. The model is discussed further below.

### 3.1 Data collection

The collection of raw data relating to symptoms and their associated diseases is an essential component in facilitating the overall functioning of the proposed system. Primarily, data related to wheat has been acquired from many sources, encompassing the websites of governmental agriculture agencies, and agricultural research institutions as well as from SOTA, the internet, farmers, and domain experts. The primary data collected from Various sources have been stored in a rudimentary database. The document comprises a total of 100 elements. The dataset consists of a total of 120 columns representing symptoms and 61 rows representing diseases.

### 3.2 Data preprocessing

First, information is compiled from various sources, including SOTA, the internet, farmers, and domain experts. The application of the Standard Knowledge Discovery in Database (KDD) process, as outlined by [60], has been utilized to extract valuable knowledge from raw data. The consolidation of redundant qualities with distinct names has been achieved through collaboration with agricultural domain experts. The concept of information gain has been employed to discover the features that have the highest significance in the classification of a certain disease. The dimensions of the data have been efficiently reduced and simplified based on the findings of the information gain analysis.

The primary objective of this study is to examine the four key input properties that characterize the symptoms associated with a specific disease, as well as the corresponding output attribute that represents the disease itself. The data cleaning technique yielded a total of 43 distinct instances, which encompassed 14 different diseases affecting wheat crops. The data that had been cleaned underwent further verification and authentication by domain experts and the University of Agriculture. The principles of diseases have been presented in Table 1.

**Table 1:** Developed principles for wheat disease.

Sr#	Part	Color	Apperance_Of_Part	WheatDisease
1	Stem	Brick_red	Long_and_narrow_streaks	Black_stem_rust
2	Stem	Black	Long_and_narrow_streaks	Black_stem_rust
3	Stem	Brick_red	Long_and_narrow_pustules	Black_stem_rust
4	Stem	Black	Long_and_narrow_pustules	Black_stem_rust
5	Leaves	Orange	Small_pustules	Leaf_rust
6	Leaves	Black	Small_pustules	Leaf_rust
7	All_green_parts	Yellow	Small_pustules	Stripe_rust
8	All_green_parts	Black	Small_pustules	Stripe_rust
9	Grain	Black	Dark_diseased_ears	Loose_smut
10	Grain	Black	Powder_smut_spores	Loose_smut
11	Grain	Black	No_grains	Loose_smut
12	Grain	Brown	Smutted_grain	Loose_smut
13	Leaves	Yellow_to_orange	Long_dark_streaks	Flag_smut
14	Grain	Black	Darker_diseased_ears	Flag_smut
15	Grain	Black	Rotten_fish_smell	Complete_bunt
16	Grain	Black	Defected_grains	Complete_bunt
17	Grain	Black	Individual_grains_infected	Partial_bunt
18	Grain	Black	Dark_flour	Partial_bunt
19	Grain	Black	fowl_smell_flour	Partial_bunt
20	Leaves	Yellow	rolled	Ear_cockle
21	Leaves	Yellow	wrinkled	Ear_cockle
22	Stem	Green	twisted	Ear_cockle
23	Grain	Yellow	distorted	Ear_cockle
24	Grain	Yellow	Gummy_and_sticky	Ear_cockle
25	Grain	Brown	Lighter_grains	Ear_cockle
26	Leaves	Yellow	rolled	Tundo
27	Leaves	Yellow	wrinkled	Tundo
28	Leaves	Yellow	dead	Tundo
29	Stem	Green	dead	Tundo
30	Grain	Light_brown	Small_nematode_galls	Tundo
31	Grain	Brown	Lighter_grains	Tundo
32	Whole_plant	Green	Small_in_size	Tundo
33	Grain	Brown	No_grains	Black_point_complex
34	Grain	Brown	Shriveled_grains	Black_point_complex
35	Grain	Black	Black_tips	Black_point_complex
36	Grain	Gray-brown	Wider_kernels	Common_bunt
37	Grain	Gray-green	Wider_kernels	Common_bunt
38	Grain	Dark-green	Black_powdery_coating	Sooty_head_molds
39	Grain	Black	Black_powdery_coating	Sooty_head_molds
40	Grain	Dark-brown	Brown_streaks	Stagonospora_nodorum_blotch
41	Grain	Purple	Brown_streaks	Stagonospora_nodorum_blotch
42	Leaves	Yellow	Flame_like	Barley_yellow_dwarf’
43	Leaves	Red	Flame_like	Barley_yellow_dwarf’

### 3.3 Feature extraction

Feature extraction holds a paramount role in the development of machine learning models. The effectiveness of machine learning algorithms is intricately tied to the selection and engineering of features. When the features extracted are pertinent to the specific objectives of the task, machine learning classifiers can achieve notably high accuracies in distinguishing between various classes.

The fundamental concept behind feature extraction is to sift through the multitude of potential data attributes and retain only those that bear substantial importance in characterizing the object of study. By identifying and prioritizing features that strongly contribute to the representation of the subject matter, this process not only enhances the model’s ability to differentiate between classes but also yields significant computational benefits.

In essence, feature extraction is a strategic curation of data attributes, favoring those with the greatest significance in describing the underlying patterns in the data. This meticulous feature selection and engineering not only elevates the predictive power of machine learning models but also streamlines the computational complexity by sparing the system from the burden of processing extraneous or less meaningful features.

Wheat disease signs and symptoms are labeled as "WheatDisease" to serve as a predictor variable in Table 1. The domain experts who validated the data agreed that these characteristics are useful in diagnosing disease. Some of these signs are also present in the literature conducted on wheat crop diseases. In this respect, for classification, all symptoms of various diseases of wheat that are most prevalent and well-known in the literature are chosen. These characteristics include the occurrence of a symptom in any region of the plant.

#### 3.3.1 Performing feature extraction with count vectorizer

Transform textual descriptions of wheat diseases into token-based vectors, creating a Bag of Words model that relies on word frequency. This transformation is achieved using the Count Vectorizer method from the Sklearn Library. Through this process, construct vectors that represent the features of the text data. Before applying the count vectorizer, it is essential to analyze high-frequency words, as they serve as a comprehensive representation of text-based wheat diseases.

#### 3.3.2 Performing feature extraction with TF-IDF (Term Frequency-Inverse Document Frequency)

Next, this study employs the Term Frequency-Inverse Document Frequency (TF-IDF) method, which involves calculating the term frequency (TF), i.e., the frequency of each word within a text-based description of a wheat disease. This process results in the creation of a vocabulary comprised of words present in the text data. This vocabulary is then utilized for encoding both visible and unseen text.

### 3.4 Data splitting

After pre-processing and feature extraction steps on the text-based datasets, the next phase involves dividing the data into training and test sets. In this context, 80% of the dataset is allocated for training purposes, while the remaining 20% is reserved for testing. This partitioning is essential for the subsequent application of classification algorithms. The training data, constituting 80% of the original dataset, serves as the input for training the classifiers. During this training phase, the ML algorithms learn patterns and relationships within the data. On the other hand, the test data, comprising the remaining 20%, is employed to evaluate the performance of the trained classifiers.

### 3.5 Classification

In the realm of Machine Learning (ML), supervised classification stands out as a foundational task, offering a rich variety of classification algorithms. For the current evaluation, four specific algorithms ‐ Decision Trees (DT), Random Forest (RF), Support Vector Machine (SVM), and AdaBoost ‐ have been selected for the proposed method for the training data to construct predictive models.

**Decision Trees**, as classifiers, embody tree-like structures defined by rules and are esteemed for their interpretability, mirroring human reasoning [61]. Widely utilized in both research and practical applications, Decision Trees, such as Quinlan’s C4.5 [62] and Classification and Regression Tree (CART) [63], hold a prominent position in the field of ML [64, 65]. The advantages of DT induction algorithms extend to robustness in handling noisy data, including managing missing values and imbalanced classes. Furthermore, they are recognized for their low computational cost and adeptness in handling redundant attributes [66].

The significance of hyper-parameter values in ML algorithms cannot be overstated, directly impacting the predictive performance of the models they generate. Researchers have dedicated extensive studies to understand the influence of hyper-parameters on various algorithms, employing techniques ranging from traditional methods like Grid Search (GS) and Random Search (RS) [67], to advanced approaches such as meta-heuristics (MTH) [68] and meta-learning (MtL) [69]. Despite the abundance of studies on hyper-parameter optimization for Support Vector Machines (SVMs) [70, 71] and Neural Networks (NNs) [72], fewer investigations have specifically targeted the optimization of hyper-parameters for DT induction algorithms [73–75].

Moving to **Random Forest**, it is defined as a classifier comprising a collection of tree-structured classifiers, each casting a unit vote for the most popular class at input x. With a large number of trees generated, the winning class is determined by the one with the most votes [76].

**Support Vector Machine**, another classifier in the evaluation, is a supervised learning model particularly effective in text categorization. This classifier establishes optimal boundaries to separate positive and negative training samples, demonstrating resilience against overfitting when provided with less noisy data [77].

**AdaBoost,** is effective in mitigating overfitting, particularly in scenarios with less noisy data, as observed in the study by [78]. However, due to its inherent binary nature, AdaBoost achieved an equivalent level of accuracy to SVM when applied to the given dataset.

The superiority of the classification methods used in this study—Decision Tree (DT), Random Forest (RF), Support Vector Machine (SVM), and AdaBoost—stems from their proven effectiveness in handling text-based data for classification tasks. These methods are particularly well-suited for text data due to their ability to handle high-dimensional features, as seen with Count Vectorization (CV) and Term Frequency-Inverse Document Frequency (TF-IDF). While recent advanced methods like deep learning are highly effective with large image datasets, they often require substantial computational resources and large volumes of labeled data, which may not always be feasible in real-world agricultural settings. The chosen methods in this study strike a balance between computational efficiency, accuracy, and applicability to diverse field conditions, providing a practical and reliable solution for wheat disease detection based on text data.

### 3.6 The dominant hyperparameter: Maximum depth’s central role

Is the maximum depth hyperparameter the most critical factor influencing the complexity and effectiveness of tree-based models? Typically, when using the CART Decision Tree algorithm, three hyperparameters (Maximum Depth, Minimum Samples Split, and Minimum Samples Leaf) are fine-tuned to find the ideal model and prevent overfitting, which can be computationally intensive [79]. To reduce the computational burden while still achieving a robust model, it’s advisable to concentrate on optimizing those specific hyperparameters that have the greatest impact on model performance.

### 3.7 Mobile application

**Fig 2:**
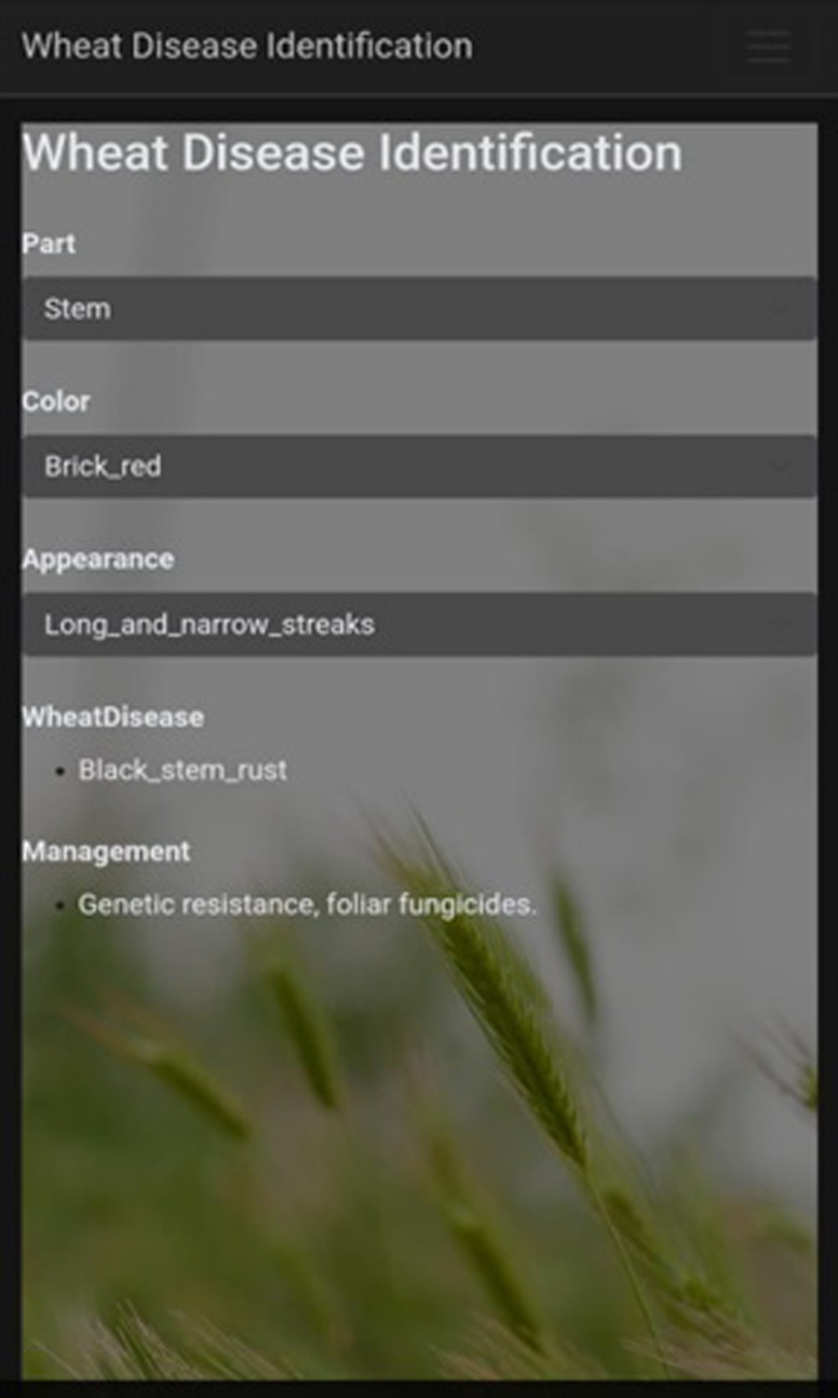
(a): User input screen.

Smartphone applications have become pivotal in enhancing accessibility and efficiency across various sectors, including agriculture. The proposed wheat disease detection system leverages the widespread use of Android smartphones to bring advanced machine learning capabilities directly to the hands of farmers. This integration is crucial for ensuring that the technological solutions developed in this study are both practical and impactful as shown in Fig 2.

The mobile application is designed to be user-friendly, allowing farmers to diagnose wheat diseases in the field with minimal effort. Users can input visual characteristics of the wheat plant, such as the color and appearance of affected areas, through an intuitive interface. For example, in one scenario, a farmer might select ’stem’ for the crop part, ’brick red’ for the color, and ’long and narrow streaks’ for the appearance. The application then processes this input using the trained machine learning models and provides an accurate diagnosis, along with recommended management strategies.

One of the key advantages of integrating smartphones into this system is the ability to perform disease detection on-site and in real-time, without the need for expensive or complex equipment. This is particularly beneficial in rural areas where access to professional agricultural services may be limited. By offering an offline mode, the application ensures that farmers can still utilize its features even in areas with poor internet connectivity, making it a reliable tool in various field conditions. During the development and deployment phases, several challenges were encountered, including issues related to data quality, model accuracy, and user interface design. These were addressed by collaborating with local experts to ensure relevant data, optimizing machine learning models for better accuracy, and designing an intuitive, user-friendly interface. Additionally, offline functionality and comprehensive training were implemented to ensure usability in areas with limited internet access.

The system’s backend, built using the sci-kit-learn ML library, processes the input data and matches it against the trained models to generate predictions. The results are then displayed on the smartphone screen, providing farmers with actionable insights in a matter of seconds. This seamless integration of smartphones into the disease detection pipeline not only streamlines the diagnostic process but also empowers farmers to make informed decisions quickly, ultimately leading to better crop management and higher yields.

In conclusion, the integration of smartphones in this wheat disease detection system represents a significant step forward in agricultural technology. It bridges the gap between advanced machine learning models and practical, on-the-ground application, making sophisticated disease diagnosis accessible to farmers everywhere. Fig 2 illustrates the mobile application’s ability to assist farmers in diagnosing wheat diseases directly in the field with minimal effort.

### 3.8 Performance evaluation of the proposed system

The performance of the proposed system is assessed for wheat disease using a classification setup, employing the confusion matrix Table 2, specific to the disease (as provided in Table 1). Four evaluation metrics are computed using Eqs (1) through (4) based on the values in these confusion matrices. To gain an understanding of the overall performance, averages are calculated across the columns. These equations involve various values obtained from the confusion matrix of each ML model. These critical values encompass:

**Table 2:** Confusion matrix.

	Predicted Positive	Predicted Positive
**Actual Positive**	TP	FN
**Actual Negative**	FP	TN


$$(A)Accuracy=\frac{TP+TN}{TP+FN+TN+FP}$$ (1)

$$(P)Precision=\frac{TP}{TP+FP}$$ (2)

$$(R)Recall=\frac{TP}{TP+FN}$$ (3)

$$F1-Score=\frac{2\times P\times R}{P+R}$$ (4)

**True_Positive (TP):** Specifies the number of instances in which the ML model accurately identified positive samples in the testing data as positive.

**True_Negative (TN):** This indicates the frequency with which the ML model correctly classified instances belonging to the negative class.

**False_Positive (FP):** Reflects the rate at which our model incorrectly classified different positive classes.

**False_Negative (FN):** Represents the misclassification rate for different positive classes.

## 4. Result and discussion

In this section, discusses the test environment and the outcomes of classifying wheat diseases using text data, achieved through the implementation of DT, RF, SVM, and AdaBoost. This study will specifically address the accuracy of the proposed system as shown in Table 3. Here is an in-depth analysis of the effectiveness of ML models when applied to classification tasks provided. To carry out this analysis, the performance of the proposed system is assessed by examining its accuracy, precision, recall, and F1 score.

**Table 3:** Phase 1, proposed system is compared with SOTA [1] when applied to the extracted features, achieving the highest level of accuracy.

Phases	Reference	ML Classifiers	No. of Diseases																	Accuracy %
			1	2	3	4	5	6	7	8	9	10	11	12	13	14	15	16	17	
			Black Stem Rust	Leaf Rust	Stripe Rust	Loose Smut	Flag Smut	Complete Bunt	Partial Bunt	Ear Cockle	Tundo	Black Point Complex	Common Bunt	Sooty Head Molds	Stagonospora Nodorum Blotch	Barley Yellow Dwarf	Sectoral Leaf Blotch	Eusarium Head Blinght	Common Root Rot	
1	[1]	Fuzzy Logic	**✓**	**✓**	**✓**	**✓**	**✓**	**✓**	**✓**	**✓**	**✓**	**✓**	**×**	**×**	**×**	**×**	**×**	**×**	**×**	99.3
	Proposed System	DT (CV)	**✓**	**✓**	**✓**	**✓**	**✓**	**✓**	**✓**	**✓**	**✓**	**✓**	**✓**	**✓**	**✓**	**✓**	**×**	**×**	**×**	86
		DT (TF-IDF)	**✓**	**✓**	**✓**	**✓**	**✓**	**✓**	**✓**	**✓**	**✓**	**✓**	**✓**	**✓**	**✓**	**✓**	**×**	**×**	**×**	86
		RF (CV)	**✓**	**✓**	**✓**	**✓**	**✓**	**✓**	**✓**	**✓**	**✓**	**✓**	**✓**	**✓**	**✓**	**✓**	**×**	**×**	**×**	86
		RF (TF-IDF)	**✓**	**✓**	**✓**	**✓**	**✓**	**✓**	**✓**	**✓**	**✓**	**✓**	**✓**	**✓**	**✓**	**✓**	**×**	**×**	**×**	85
		SVM (CV)	**✓**	**✓**	**✓**	**✓**	**✓**	**✓**	**✓**	**✓**	**✓**	**✓**	**✓**	**✓**	**✓**	**✓**	**×**	**×**	**×**	99
		SVM (TF-IDF)	**✓**	**✓**	**✓**	**✓**	**✓**	**✓**	**✓**	**✓**	**✓**	**✓**	**✓**	**✓**	**✓**	**✓**	**×**	**×**	**×**	89
		AdaBoost (CV)	**✓**	**✓**	**✓**	**✓**	**✓**	**✓**	**✓**	**✓**	**✓**	**✓**	**✓**	**✓**	**✓**	**✓**	**×**	**×**	**×**	91
		AdaBoost (TF-IDF)	**✓**	**✓**	**✓**	**✓**	**✓**	**✓**	**✓**	**✓**	**✓**	**✓**	**✓**	**✓**	**✓**	**✓**	**×**	**×**	**×**	89
2	[3]	DT	**✓**	**×**	**✓**	**✓**	**×**	**×**	**×**	**×**	**×**	**✓**	**✓**	**✓**	**×**	**×**	**✓**	**✓**	**✓**	98
	Proposed System	DT (CV)	**✓**	**✓**	**✓**	**✓**	**✓**	**✓**	**✓**	**✓**	**✓**	**✓**	**✓**	**✓**	**✓**	**✓**	**×**	**×**	**×**	86
		DT (TF-IDF)	**✓**	**✓**	**✓**	**✓**	**✓**	**✓**	**✓**	**✓**	**✓**	**✓**	**✓**	**✓**	**✓**	**✓**	**×**	**×**	**×**	86
		RF (CV)	**✓**	**✓**	**✓**	**✓**	**✓**	**✓**	**✓**	**✓**	**✓**	**✓**	**✓**	**✓**	**✓**	**✓**	**×**	**×**	**×**	86
		RF (TF-IDF)	**✓**	**✓**	**✓**	**✓**	**✓**	**✓**	**✓**	**✓**	**✓**	**✓**	**✓**	**✓**	**✓**	**✓**	**×**	**×**	**×**	85
		SVM (CV)	**✓**	**✓**	**✓**	**✓**	**✓**	**✓**	**✓**	**✓**	**✓**	**✓**	**✓**	**✓**	**✓**	**✓**	**×**	**×**	**×**	99
		SVM (TF-IDF)	**✓**	**✓**	**✓**	**✓**	**✓**	**✓**	**✓**	**✓**	**✓**	**✓**	**✓**	**✓**	**✓**	**✓**	**×**	**×**	**×**	89
		AdaBoost (CV)	**✓**	**✓**	**✓**	**✓**	**✓**	**✓**	**✓**	**✓**	**✓**	**✓**	**✓**	**✓**	**✓**	**✓**	**×**	**×**	**×**	91
		AdaBoost (TF-IDF)	**✓**	**✓**	**✓**	**✓**	**✓**	**✓**	**✓**	**✓**	**✓**	**✓**	**✓**	**✓**	**✓**	**✓**	**×**	**×**	**×**	89

### 4.1 Test environment

The test experimental setup that underlies the study contribution is characterized by specific information about the system, datasets, and machine learning algorithms utilized. Here are the comprehensive details: The experimentation is conducted on a 64-bit Windows 10 Enterprise platform. The central processing unit employed for this study is an Intel(R) Core(TM) i5-7200U CPU running at a base clock speed of 2.50 GHz, with a maximum turbo frequency of 2.71 GHz. The experimental system is equipped with 8.0 gigabytes (GB) of Random Access Memory (RAM). The primary machine learning classifiers employed in this study are the DT, RF, SVM, and AdaBoost Classifiers. These classifiers are pivotal in the study as they form the basis for the analysis and classification of data. In addition to this, the proposed system leverages various libraries for its implementation. These include Python 3.11 as the primary programming language. For the training and testing of various machine learning models, utilizing sci-kit-learn ML library version 1.3.0. Furthermore, employ the Matplotlib library, version 3.7.1, a Python-based tool for visualizing a wide range of content, such as images, results, and graphs.

### 4.2 Decision tree

**Fig 3:**
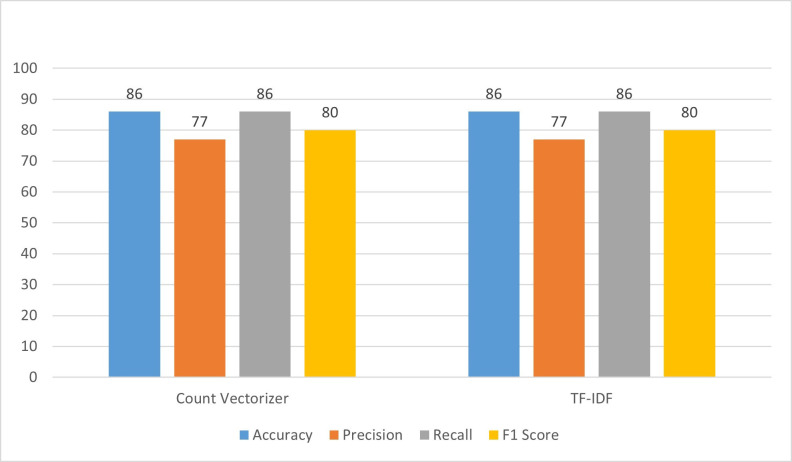
Performance evaluation of DT.

In the first experiment in Fig 3, Decision Trees (DT) are employed with a count vectorizer, incorporating specific hyperparameters such as max_depth = 10, min_samples_split = 3, and min_samples_leaf = 1. The results revealed an accuracy of 86%, indicating that the model correctly predicted the outcome in 86% of cases. Precision, which measures the accuracy of positive predictions, stood at 77%, implying that out of all instances predicted as positive, 77% are indeed positive. The recall, which gauges the model’s ability to identify all relevant instances, is 86%, signifying that the model captured 86% of all actual positive instances. The F1 score, a balanced metric considering both precision and recall, is 80%. Moving on to the second experiment, TF-IDF (Term Frequency-Inverse Document Frequency) is utilized without the incorporation of specific hyperparameters. Despite the absence of hyperparameter tuning, the accuracy remained at 86%, aligning with the first experiment. Precision, recall, and F1_score are also consistent at 77%, 86%, and 80%, respectively. This suggests that the TF-IDF approach, without hyperparameter adjustments, yielded comparable performance to the count vectorizer with carefully selected hyperparameters in the first experiment. In essence, both experiments resulted in similar performance metrics, highlighting the robustness of the models in achieving a balanced accuracy, precision, recall, and F1 score.

### 4.3 Random forest

**Fig 4:**
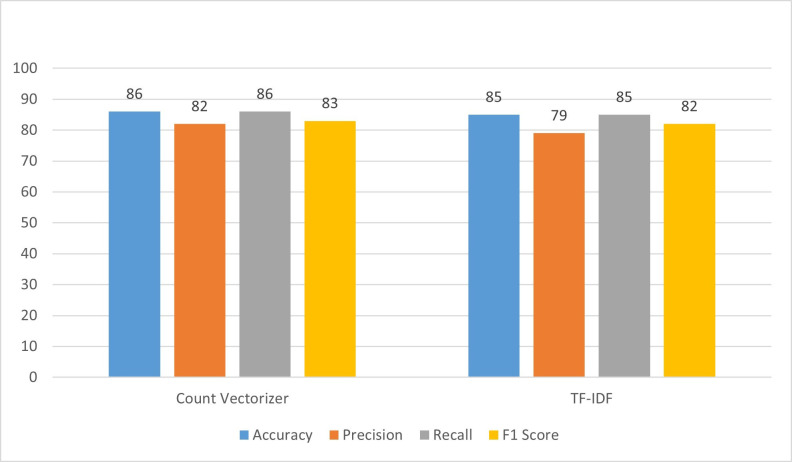
Performance evaluation of RF.

In the initial experiment, the outcomes are presented in Fig 4, the Random Forest (RF) is employed with a count vectorizer. The results indicated an accuracy of 86%, denoting that the model made correct predictions in 86% of cases. Precision, which reflects the accuracy of positive predictions, is at 82%, signifying that 82% of instances predicted as positive are indeed positive. The recall, measuring the model’s ability to identify all relevant instances, stood at 86%, indicating that the model captured 86% of all actual positive instances. The F1_score, a balanced metric considering both precision and recall, is calculated at 83%. Moving on to the second experiment, TF-IDF is utilized, and specific hyperparameters (max_depth = none, min_samples_split = 2) are employed. The results of this experiment revealed that accuracy is 85% when compared to the count vectorizer in the first experiment. Precision is 79%, recall is 85%, and the F1 score is 82%. In summary, the shift from count vectorizer to TF-IDF, along with the alteration of hyperparameters, resulted in a notable decrease in accuracy and performance metrics. The accuracy dropped to 85%, and there was a reduction in precision, recall, and the F1 score. This suggests that the choice of vectorization method (count vectorizer or TF-IDF) and the fine-tuning of hyperparameters significantly impact the model’s predictive capabilities.

### 4.4 Support Vector Machine

**Fig 5:**
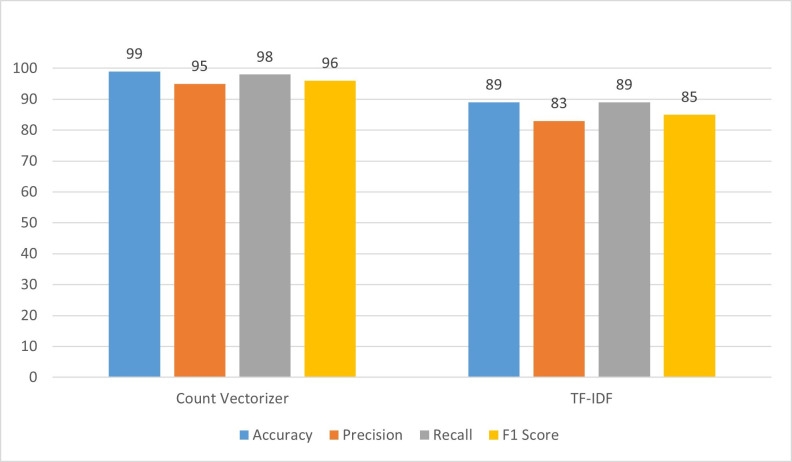
Performance evaluation of SVM.

In Fig 5, a Support Vector Machine (SVM) is applied using the count vectorizer in the first experiment, and the results demonstrated a high level of performance. The accuracy reached 99%, indicating that the model correctly predicted outcomes in 99% of cases. Precision, which gauges the accuracy of positive predictions, is notably high at 95%, signifying that almost all instances predicted as positive are indeed positive. However, the recall, representing the model’s ability to identify all relevant instances, is 98%, implying that the model captured 98% of all actual positive instances. The F1_score, a balanced metric considering both precision and recall, is calculated at 96%. In the second experiment, TF-IDF is employed instead of the count vectorizer. However, the results showed a significant decrease in performance compared to the first experiment. The accuracy dropped to 89%, indicating a lower overall correctness in predictions. Precision decreased to 83%, meaning that the proportion of correctly predicted positive instances decreased. The recall also dropped to 89%, suggesting that the model captured a lower percentage of actual positive instances. Consequently, the F1 score, taking into account both precision and recall, declined to 85%. In summary, the transition from count vectorizer to TF-IDF led to a notable decrease in SVM’s performance metrics, including accuracy, precision, recall, and F1 score. This emphasizes the sensitivity of SVM to the choice of vectorization method, and in this case, the count vectorizer proved more effective in capturing patterns in the data.

### 4.5 AdaBoost

**Fig 6:**
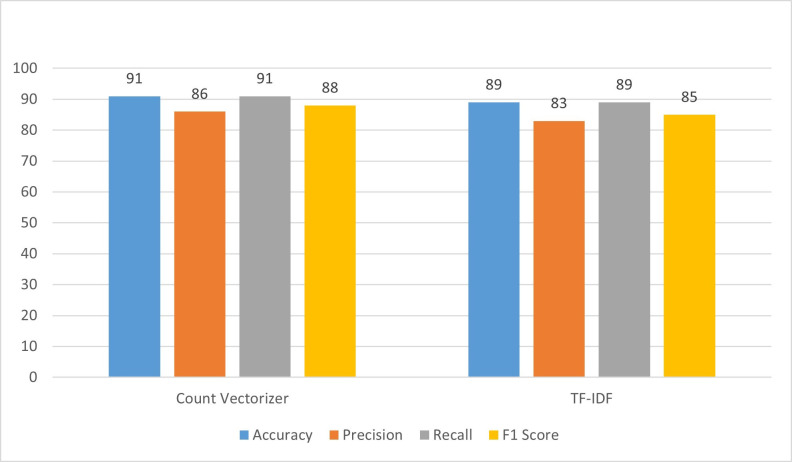
Performance evaluation of AdaBoost.

In the first experiment, AdaBoost is employed with a count vectorizer, and the results are summarized in Fig 6. The findings reveal that the accuracy of the model reached 91%, signifying that it made correct predictions in 91% of cases. Precision, which measures the accuracy of positive predictions, stood at 86%, indicating that 86% of instances predicted as positive are indeed positive. The recall, representing the model’s ability to identify all relevant instances, is 91%, implying that the model captured 91% of all actual positive instances. The F1_score, a balanced metric considering both precision and recall, is calculated at 88%. Moving on to the second experiment, TF-IDF is employed instead of the count vectorizer. However, the results of this experiment indicated a decrease in performance compared to the first experiment. The accuracy dropped to 89%, suggesting lower overall correctness in predictions. Precision decreased to 83%, indicating a reduction in the proportion of correctly predicted positive instances. The recall also dropped to 89%, implying that the model captured a lower percentage of actual positive instances. Consequently, the F1 score, considering both precision and recall, declined to 85%. To summarize, the transition from the count vectorizer to TF-IDF led to a noticeable decrease in AdaBoost’s performance metrics, including accuracy, precision, recall, and F1_score. This highlights the sensitivity of AdaBoost to the choice of vectorization method, and in this case, the count vectorizer proved more effective in capturing patterns in the data.

**Fig 7:**
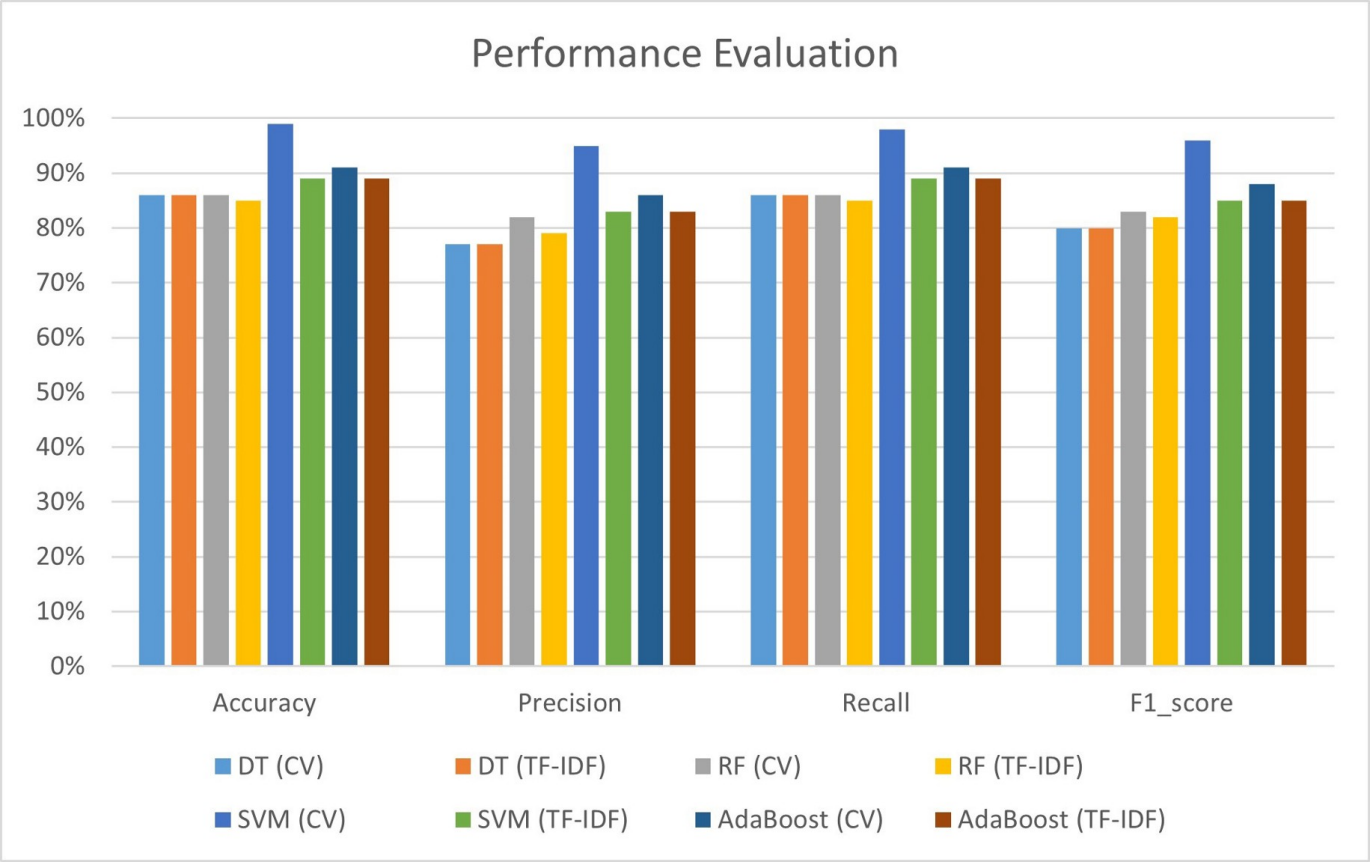
Results of all experiments with count vectorizer and TF-IDF with hyperparameter (Compare with SOTA).

DT, SVM, RF, and AdaBoost are examined to determine how vectorization strategies affect model performance in Fig 7. Decision Trees performed consistently in count vectorization and TF-IDF, with stable accuracy, precision, recall, and F1_score. It appears that DT is not sensitive to the vectorization approach. However, SVM is sensitive to vectorization, losing accuracy, precision, and F1_score when switching from count vectorizer to TF-IDF. This emphasizes the need for careful SVM vectorization method selection. Random Forest, like SVM, performed poorly with TF-IDF. Although less significant than in SVM, the pattern shows that count vectorization may benefit Random Forest in this scenario. AdaBoost also performed worse with TF-IDF, supporting the concept that the vectorization approach may affect SVM, RF, and AdaBoost. Beyond vectorization, hyperparameter adjustment affected SVM. SVM may need to fine-tune hyperparameters to optimize TF-IDF performance. In SVM, TF-IDF significantly reduced recall, demonstrating the precision-recall trade-off. This trade-off emphasizes the need to examine the analysis’s goals and the right balance between properly anticipating positive instances and capturing all relevant examples.

This study suggests that model performance can vary between algorithms, hence the vectorization method and hyperparameters should be adjusted to the dataset and analytic goals. These insights help us evaluate algorithmic performance and make informed decisions when applying machine learning to real-world challenges.

The discussion highlights the effectiveness of the proposed system in wheat disease detection compared to existing state-of-the-art (SOTA) approaches as shown in Table 3 While previous methods like Fuzzy Logic [1] and Decision Trees [3] demonstrated high accuracy (99.3% and 98%, respectively) in classifying a limited number of diseases, the proposed system excels by accurately identifying a broader spectrum of 14 wheat diseases. For instance, Decision Trees achieved 86% accuracy with both Count Vectorizer (CV) and Term Frequency-Inverse Document Frequency (TF-IDF), while Random Forest showed similar robustness with accuracies of 86% (CV) and 85% (TF-IDF). The Support Vector Machine (SVM) classifier was particularly effective, reaching 99% accuracy with CV, though slightly lower at 89% with TF-IDF. AdaBoost also performed well, achieving 91% accuracy with CV and 89% with TF-IDF.

The comparison suggests that while traditional methods perform well in specific contexts, the proposed system’s ability to handle more complex datasets with multiple diseases is a significant advantage. However, the discussion would benefit from a deeper comparison with recent literature, particularly concerning advanced methods like fuzzy logic and deep learning or hybrid approaches.

Additionally, the integration of smartphone applications, although briefly mentioned, plays a crucial role in the system’s practical utility. The application, designed for ease of use with features like language support and offline functionality, allows farmers to quickly and accurately identify wheat diseases, even in regions with limited access to advanced agricultural services. By combining machine learning with a user-friendly smartphone interface, the proposed system not only improves disease detection accuracy but also enhances its accessibility and practicality in real-world agricultural settings, ultimately supporting better decision-making and crop management.

## 5. Conclusions

This study introduced a system for the identification of wheat diseases using the ML approach and used a text base dataset comprising various types of wheat diseases, such as black stem rust, leaf rust, stripe rust, loose smut, flag smut, complete bunt, partial bunt, ear cockle, tundo, black point complex, common bunt, sooty head molds, stagonospora nodorum blotch, and barley yellow dwarf. Feature selection techniques are employed to ensure precise preprocessing. Two features are extracted using the count vectorizer and TF-IDF. Four machine learning models are trained using extracted characteristics to conduct a comparison of the performance of the SOTA ML model. Following the comparative analysis in Table 3, it has been discovered that the proposed system exhibits better accuracy as it successfully predicted 14 different wheat diseases with management. The effectiveness of the proposed system is assessed using a text-based dataset and a diverse set of evaluation metrics, including accuracy, precision, recall, and F1-score. To facilitate a comprehensive assessment, a comparative analysis is undertaken between the proposed system and the current SOTA methodologies. Consequently, the proposed system demonstrates a higher level of accuracy in the identification of 14 different wheat diseases compared to SOTA methodologies. The future goal of this study is to expand the existing dataset to include more classes of wheat diseases as well as other crops and deployed in various regions outside the Pakistan, also integrate a treatment suggestion feature for the found diseases. This implementation would enable effective and timely assistance to farmers in the field while minimizing resource and time wastage on a global scale.
